# Pericytes as Key Players in Retinal Diseases: A Comprehensive Narrative Review

**DOI:** 10.3390/biology14070736

**Published:** 2025-06-20

**Authors:** Fabiana D’Esposito, Francesco Cappellani, Federico Visalli, Matteo Capobianco, Lorenzo Rapisarda, Alessandro Avitabile, Ludovica Cannizzaro, Roberta Malaguarnera, Giuseppe Gagliano, Antonino Maniaci, Mario Lentini, Giuseppe Montalbano, Mohamed Amine Zaouali, Dorra H’mida, Giovanni Giurdanella, Caterina Gagliano

**Affiliations:** 1 Department of Neurosciences, Reproductive Sciences and Dentistry, University of Naples Federico II, Via Pansini 5, 80131 Napoli, Italy; f.desposito@imperial.ac.uk; 2 Imperial College Ophthalmic Research Group (ICORG) Unit, Imperial College, 153-173 Marylebone Rd, London NW1 5QH, UK; 3 Department of Medicine and Surgery, University of Enna “Kore”, Piazza dell’Università, 94100 Enna, Italy; francesco.cappellani@unikore.it (F.C.); roberta.malaguarnera@unikore.it (R.M.); giovanni.giurdanella@unikore.it (G.G.); caterina.gagliano@unikore.it (C.G.); 4 Mediterranean Foundation “G.B. Morgagni”, 95125 Catania, Italy; 5 Department of Ophthalmology, University of Catania, 95123 Catania, Italycapobiancoteo@gmail.com (M.C.);; 6 ASP 8 Siracusa, PO Umberto I, 96100 Siracusa, Italy; 7 Faculty of Medicine, University of Catania, 95123 Catania, Italy; 8 ENT Unit, Department of Surgery, Asp 7 Ragusa, 97100 Ragusa, Italy; 9 Zebrafish Neuromorphology Laboratory, Department of Veterinary Sciences, University of Messina, 98168 Messina, Italy; gmontalbano@unime.it; 10 Laboratory of Human Genome and Multifactorial Diseases (LR12ES07), Faculty of Pharmacy, University of Monastir, Avicenne Street, Monastir 5019, Tunisia; 11 Department of Cytogenetics and Reproductive Biology, Farhat Hached Hospital, Sousse 4021, Tunisia

**Keywords:** pericytes, retina, blood–retinal barrier, diabetic retinopathy, age-related macular degeneration, glaucoma, retinal vein occlusion, vascular dysfunction, pericyte-targeted therapy

## Abstract

Pericytes, specialized mural cells surrounding microvessels, play a crucial role in maintaining vascular homeostasis and function across various organs, including the eye. These versatile cells regulate blood flow, support the integrity of the blood–retinal barrier, and contribute to angiogenesis. Recent advancements in molecular and cellular biology have revealed the heterogeneity of pericytes and their critical involvement in ocular physiology and pathology. This review provides a comprehensive analysis of pericyte functions in ocular health and their implications in diseases such as diabetic retinopathy, age-related macular degeneration, glaucoma, and retinal vein occlusion. Pericyte dysfunction is implicated in vascular instability, neurovascular coupling failure, inflammation, and pathological neovascularization, contributing to vision-threatening disorders. The review further explores recent findings on pericyte-targeted therapies, including pharmacological agents, gene therapy, and cell-based approaches, aiming to restore pericyte function and preserve ocular health.

## Simple Summary

Vision loss caused by retinal diseases such as diabetic retinopathy, age-related macular degeneration, glaucoma, and retinal vein occlusion has a significant impact on quality of life and remains a major global health concern. Although these diseases are common, many questions remain about how they develop and progress. Recent studies have highlighted the critical role of pericytes—specialized cells that support and stabilize tiny blood vessels—in maintaining the health of the eye. Pericyte damage or loss is now recognized as an early and important event in many retinal disorders, leading to inflammation, leaky vessels, and harmful new vessel growth. This review explores how pericytes contribute to both normal eye function and disease, examining the cellular and molecular processes involved. It also looks ahead to future treatments that aim to protect or replace these cells, including promising approaches using drugs, gene therapy, and cell-based strategies. Better understanding of pericyte biology could help improve early diagnosis and lead to more effective therapies to preserve vision.

## 1. Introduction

Pericytes, the mysterious mural cells that enclose the microvessel endothelium, have become important modulators of vascular homeostasis and function in a number of different organs, including the eye. These multifunctional cells are essential for controlling blood flow, preserving the integrity of the blood–retinal barrier, and modifying angiogenesis in ocular tissues. They are distinguished by their unique morphology and advantageous perivascular position [1]. The amazing variability and adaptability of pericytes have been revealed by recent advances in cellular and molecular biology techniques, challenging long-held beliefs about their roles and providing fresh insights into ocular physiology and pathology [2]. The eye is a perfect model system for researching pericyte biology and its consequences in health and disease because of its complex circulatory networks and special immunological capabilities. With a pericyte-to-endothelial cell ratio of roughly 1:1, the retina has a remarkably high density of pericytes compared with most other organs [3]. Their abundance in the retina, combined with evidence showing that pericyte loss leads to microvascular dysfunction, blood–retinal barrier breakdown, and vision-threatening pathologies, underscores their essential role in maintaining retinal homeostasis and visual function [4]. The blood–retinal barrier, an essential interface that controls the exchange of molecules between the blood and the neuronal retina, is formed and maintained in part by pericytes found in the retina. Notably, while the blood–brain barrier also has high pericyte coverage, its typical endothelial/pericyte ratio ranges from approximately 1:1 to 3:1, suggesting that the retina possesses one of the densest pericyte networks in the body [5]. In order to create tight junctions, regulate vascular permeability, and preserve the delicate balance of the retinal milieu, pericytes work in concert with endothelial cells through complex cell-to-cell interactions and the production of different signaling molecules [6]. The diverse functions of pericytes in the control of ocular blood flow have been highlighted by recent investigations. Researchers have shown that retinal pericytes contain contractile characteristics, which enable them to adjust blood flow and capillary diameter in response to changes in brain activity and metabolic demands [7]. Neurovascular coupling, the dynamic regulation of blood flow, is crucial for preserving retinal function and adjusting to shifting visual stimuli [8]. Pericytes have also been linked to the autoregulation of retinal blood flow, which is an essential process that keeps perfusion steady even in the face of variations in systemic blood pressure [9].

The role that pericytes play in the development of ocular angiogenesis has attracted a lot of interest lately. Pericytes are essential for the stabilization, maturation, and pruning of vessels during retinal vascular development [10]. They regulate the balance between vessel growth and regression by secreting pro- and anti-angiogenic substances such as angiopoietin-1 and vascular endothelial growth factor (VEGF) [11]. Disturbance of this delicate equilibrium in pathological situations can result in aberrant angiogenesis, as documented in wet age-related macular degeneration and proliferative diabetic retinopathy [12]. According to newly available data, pericytes have the ability to modulate the immune system, supporting the eye’s immunological privilege and inducing inflammatory reactions in the event of ocular disorders [13]. It has been demonstrated that pericytes contain a variety of immune-related molecules, including as toll-like receptors and major histocompatibility complex (MHC) class II antigens, which allow them to interact with immune cells and control local inflammatory responses [14]. Pericytes’ roles in ocular health and illness are further complicated by their immunoregulatory function.

The field of ocular pericytes has shown unanticipated heterogeneity due to the development of single-cell RNA sequencing and improved lineage tracing tools [15]. The idea that pericytes are a monomorphic cell population has been called into question by recent research that has found multiple subpopulations of pericytes in various eye areas, each with their own functional characteristics and molecular markers [16]. In addition to their functional diversity, studies suggest that pericytes in different regions of the body may arise from distinct embryonic lineages. Notably, CNS-associated pericytes—including those in the retina—have been shown to originate at least in part from the neural crest, whereas pericytes in peripheral organs are typically derived from mesodermal progenitors. This developmental heterogeneity may contribute to region-specific differences in pericyte function and response to injury or disease [17,18,19]. The varied and occasionally contradictory roles attributed to pericytes in different ocular tissues and disease states may be explained by this heterogeneity. Many eye conditions, including age-related macular degeneration, glaucoma, and diabetic retinopathy, have been linked to pericyte dysfunction [20]. One of the first noticeable changes in diabetic retinopathy is pericyte loss, which eventually causes microaneurysm development, vascular instability, and the collapse of the blood–retinal barrier [21]. Autophagy dysregulation and oxidative stress-induced apoptosis are two unique mechanisms that underlie pericyte loss in diabetes that have been clarified by recent investigations [22]. Changes in pericyte–endothelial cell interactions have been connected to the development of choroidal neovascularization and age-related macular degeneration [23]. Additionally, new research indicates that pericyte dysfunction may influence optic nerve head blood flow and perhaps exacerbate retinal ganglion cell death, which is a vascular component of glaucoma pathogenesis [24]. Developing treatment approaches that specifically target pericytes has gained traction as these cells are becoming more widely acknowledged as important participants in ocular physiology and pathology. Numerous strategies, such as pericyte transplantation, pharmacological pericyte function regulation, and gene therapy focusing on pericyte-specific pathways, have been investigated in recent preclinical research [25]. Even while these tactics appear promising in animal models, there is still much work to be done in order to translate these discoveries into therapeutic applications. It is becoming more and more evident that pericytes are much more than just supporting players in the ocular vasculature as our knowledge of their biology in the eye expands. Their multifaceted roles, which include immunomodulation and vascular stabilization, place them at the forefront of preserving ocular health and make them viable targets for treatment in a variety of eye conditions. The goal of this thorough review is to summarize the most recent research on the biology of pericytes in the eye, investigate their functions in different ocular illnesses, and talk about the prospective applications of pericyte-targeted treatments in ophthalmology.

## 2. Materials and Methods

### 2.1. Literature Search Approach

To carry out this thorough narrative review, we used a methodical and exacting approach to data synthesis and the literature search on the function of pericytes in ocular disorders. We performed a literature review through different electronic databases such as PubMed, Embase, Web of Science, and Scopus. We combined free-text keywords pertaining to pericytes and other eye disorders with Medical Subject Headings (MeSHs) phrases. To ensure the currency of our review, we primarily focused on literature published within the last decade, with particular emphasis on studies from the past five years. The search terms included the following Mesh terms: “pericytes,” “mural cells,” “retina,” “choroid,” “eye,” “ocular,” “diabetic retinopathy,” “age-related macular degeneration,” “glaucoma,” “retinopathy of prematurity,” and “uveitis”. However, when it was thought necessary to provide context or historical perspective, fundamental studies and key publications from periods other than this one were also included.

### 2.2. Inclusion and Exclusion Criteria

We included original research articles, meta-analyses, systematic reviews, and high-quality narrative reviews published in peer-reviewed journals. Studies were considered if they investigated the role of pericytes in ocular diseases and were based on human data, in vivo animal models, or in vitro experiments. Relevant articles that provided mechanistic insights, therapeutic implications, or quantifiable findings related to pericyte biology were prioritized.

We excluded single-case reports and small case series unless they presented highly novel or relevant findings. Publications that did not focus on ocular pericytes or their role in retinal pathology, or that lacked sufficient methodological detail, were also excluded. Articles not written in English were also excluded. To improve consistency and minimize bias, articles were reviewed and screened by multiple authors. Disagreements were resolved through discussion and consensus.

### 2.3. Study Selection Process and Data Extraction and Synthesis

To find possibly relevant publications, two separate reviewers went through the titles and abstracts of the first search results. After that, full-text evaluations of the chosen papers were conducted to ascertain their suitability for inclusion in the review. When there were disagreements among reviewers, they were settled by consensus and discussion. A third reviewer was consulted as needed. A standardized form was used to extract data, which included important details including the study’s design, sample size, methodology, primary findings, and limitations. After the data were extracted, they were qualitatively analyzed, with special focus on emergent themes, contradictory findings, and knowledge gaps. Even though there were no direct human or animal participants in this study, we closely examined the included studies’ ethical practices. We made sure that all human studies had informed permission and the necessary ethical approval, and that all animal studies followed the applicable regulations for the humane treatment of research animals. We are aware of the possible biases in our review process, such as publication bias and language bias (because we included only papers written in English). We thoroughly searched the gray literature and spoke with subject-matter experts to find any pertinent unpublished or ongoing studies to reduce these biases.

## 3. Diabetic Retinopathy and Pericyte Loss

One of the main causes of vision loss in the globe, diabetic retinopathy (DR), is characterized by progressive microvascular damage to the retina. One of the most important and early events in the etiology of DR is generally acknowledged to be pericyte loss [26].

Notably, pericyte loss has been well documented in retinal specimens from diabetic patients as well as in numerous diabetic animal models [27,28,29,30,31]. In DR, pericyte loss shifts the endothelial cell-to-pericyte ratio from approximately 1:1 in healthy retinas to ~4:1 [32].

Numerous studies and models indicate that pericyte dropout is among the earliest changes in the diabetic microvasculature [33]. In fact, reducing pericyte density alone (e.g., via PDGF-B gene ablation) can trigger microvascular abnormalities reminiscent of DR, suggesting pericyte loss itself can causally drive retinal changes [34]. Pericyte depletion destabilizes capillaries, leading to endothelial dysfunction, capillary leakage, and microaneurysm formation. This has led many to view pericyte loss as a primary instigator of DR’s vascular damage. However, there is active debate whether pericyte loss truly comes first or if it is a secondary consequence of prior endothelial injury and BRB breakdown. Alternative hypotheses propose that initial insults—such as hyperglycemia-induced endothelial cell dysfunction, inflammation, or tight-junction breakdown—may precede pericyte dropout [35,36]. Novel processes underlying pericyte dysfunction and depletion in the diabetic retina have been clarified by recent investigations. Chronic hyperglycemia causes oxidative stress, which in turn activates nuclear factor-κB (NF-κB) and increases the development of advanced glycation end products (AGEs), both of which lead to pericyte death [37]. Moreover, it has been demonstrated that pericytes’ heightened vulnerability to apoptosis is partly due to hyperglycemia-induced mitochondrial malfunction [38]. Vascular instability, microaneurysm development, and increased blood–retinal barrier (BRB) permeability are caused by the loss of pericytes [39]. MicroRNAs have been implicated in controlling pericyte survival and function in DR. For example, it has been observed that increased miR-195 promotes pericyte death by targeting Sirtuin 1 (SIRT1) in the diabetic retina [40]. On the other hand, it has been demonstrated that miR-126 protects retinal pericytes by blocking the p38 MAPK/NF-κB signaling pathway [41]. A critical mechanism for pericyte recruitment and survival, platelet-derived growth factor (PDGF) signaling, has also been connected to changes in pericyte–endothelial cell interactions in diabetic kidney disease [42]. In addition to outright cell death, recent evidence suggests that pericyte dysfunction and departure from the vessel wall are important factors in DR. Pericytes can detach and migrate away from capillaries in response to diabetes-related stress. This process is regulated by angiopoietin/Tie2 signaling and may be influenced by autophagy pathways. In healthy vessels, pericyte-derived angiopoietin-1 (Ang-1) activates Tie2 receptors on endothelial cells, promoting stable EC–pericyte interactions. In DR, however, the balance shifts: stressed endothelial cells upregulate angiopoietin-2, an antagonist of Ang-1, which destabilizes the EC–pericyte coupling and promotes pericyte dropout [43,44]. The loss of pericytes has profound structural and functional consequences for the retinal microvasculature. Histologically, diabetic retinal capillaries develop “pericyte ghosts,” which are empty basement membrane sleeves left behind after pericytes die or detach. These ghost outlines, along with completely acellular capillaries, are classic early lesions in diabetic retinopathy, reflecting the absence of both pericytes and endothelial cells in affected capillaries. Such changes compromise the integrity of the retinal vessels. Without pericyte support, capillaries become weak and hyperpermeable, leading to the formation of microaneurysms and the leakage of fluid and blood components [45,46]. Moreover, new data implies that cellular trans-differentiation may play a role in pericyte loss in DR in addition to apoptosis. Certain retinal pericytes change phenotypically to resemble fibroblast-like cells, which contributes to vascular fibrosis and decreased vessel functionality, according to recent lineage-tracing research in diabetic mice [47]. This pericyte-to-fibroblast transition offers prospective new targets for therapy and reflects a novel mechanism of pericyte loss in DR. With the development of sophisticated imaging methods like adaptive optics scanning laser ophthalmoscopy, diabetic patients may now see their retinal pericytes in vivo, which offers fresh perspectives on the temporal and spatial patterns of pericyte loss in human DR [48]. The development of pericyte-targeted therapies for DR, such as small-molecule inhibitors of apoptotic pathways and cell-based therapies targeted at replenishing the pericyte population, is being made possible by these technological advancements in conjunction with ongoing research into the molecular mechanisms of pericyte dysfunction [49]. Current clinical management of diabetic retinopathy primarily revolves around systemic glycemic control and anti-angiogenic therapies, including widely used intravitreal anti-VEGF agents. New dual-action biologics such as faricimab, which targets both VEGF-A and angiopoietin-2, show promise in enhancing vascular stability and potentially supporting pericyte–endothelial interactions [50]. Modulation of the angiopoietin–Tie2 axis is a compelling therapeutic strategy; exogenous administration or endogenous induction of Ang-1 could enhance vessel stabilization, while the suppression of Ang-2 expression may prevent pericyte detachment. Similarly, promoting pericyte recruitment via the PDGF-β/PDGFR-β signaling pathway—impaired in chronic hyperglycemia—offers a route to reestablish pericyte coverage and restore microvascular integrity. Animal studies suggest that interventions at this level, even during early non-proliferative stages, may reverse pericyte migration or bridging behavior and prevent irreversible vascular loss. In parallel, therapies aimed at enhancing endothelial progenitor cell (EPC) function are gaining attention, given their potential to support vascular repair in DR, where EPC deficits are commonly observed [51].

The integrity of the inner blood–retinal barrier—largely maintained by tightly coupled endothelial cells with pericyte support—poses a major challenge for delivering drugs to the retina. In early DR (or in health), an intact BRB severely limits systemic drug penetration into retinal tissue: as little as ~1–2% of the circulating drug may reach the vitreous and retina [52]. This protective barrier, while crucial for neural homeostasis, means that therapeutic agents have difficulty accessing targets like retinal pericytes when the BRB is intact. Consequently, current treatments for DR (e.g., anti-VEGF) often rely on intravitreal injection to bypass the BRB entirely.

Hypothetically, transient or disease-induced BRB disruption could offer a therapeutic window to enhance drug penetration; in disease states associated with BRB compromise, such as diabetic macular edema or uveitis, permeability changes may also allow nanocarrier-based systems to reach intra-retinal targets more effectively [53]. One strategy even proposes exploiting a transient, controlled blood–ocular barrier breakdown to improve systemic drug delivery to the retina [54]. In advanced DR where BRB disruption is present, systemically administered therapies might gain improved access to diseased capillaries and pericytes that were previously secluded behind a tight barrier.

## 4. Age-Related Macular Degeneration and Pericyte Dysfunction

The progressive deterioration of the retinal pigment epithelium (RPE), photoreceptors, and choroidal vasculature characterizes age-related macular degeneration (AMD), one of the main causes of permanent visual loss in older persons. Though less research has been performed on pericytes’ role in AMD pathogenesis than on diabetic retinopathy, new data indicates that pericyte dysfunction plays a major role in the onset and progression of AMD [55]. In AMD, there are major changes to the choroid, a highly vascularized region that provides oxygen and nutrients to the outer retina. These changes include aberrant neovascularization and vascular atrophy. The innermost layer of the choroid, the choriocapillaris, depends on choroidal pericytes to maintain its structural and functional integrity, as evidenced by recent research [56]. The structural alterations and decreased blood vessel coverage exhibited by choroidal pericytes in early AMD may be responsible for the collapse of the outer blood–retinal barrier and the formation of drusen, which is a characteristic feature of this stage of the disease [57]. Advanced AMD is characterized by intricate interactions between pericytes, endothelial cells, and the extracellular matrix in the surrounding tissue. Recent ultrastructural studies have demonstrated that pericyte degeneration occurs in the choriocapillaris of AMD eyes, particularly in the early stages, and may precede endothelial cell dropout. Aberrant, dysfunctional, and dying pericytes were found in a majority of capillaries, even in regions where endothelial fenestrations remained intact, suggesting that pericyte loss may initiate vascular instability and contribute to choroidal atrophy in geographic atrophy [58]. Further adding to the inflammatory environment that characterizes AMD is the loss of pericytes in the choroid, which has been linked to the buildup of complement components and the activation of the alternative complement pathway [59]. Neovascular AMD, also called wet AMD, is caused by aberrant blood vessel growth from the choroid into the subretinal region. This abnormal blood vessel growth causes fluid buildup, bleeding, and finally, scarring. The function of pericytes in this abnormal angiogenesis process has been clarified by recent research. Pericytes normally restrict the growth of endothelial cells and the sprouting of new vessels. Nevertheless, this regulatory role seems to be weakened in the context of AMD [60]. Studies have demonstrated that pericytes inside the aged choroid display modified expression of important angiogenic factors, including platelet-derived growth factor-B (PDGF-B) and VEGF, upsetting the delicate equilibrium between pro- and anti-angiogenic signals [61]. Moreover, pericytes have been connected to the development and maintenance of lesions known as choroidal neovascularization (CNV). Pericyte recruitment to newly created capillaries occurs rapidly and is regulated by the CXCR4/SDF-1 axis, suggesting a possible therapeutic target for reducing CNV progression [62]. This was proven in a recent work employing a laser-induced CNV model in mice. The development of sophisticated imaging methods like optical coherence tomography angiography (OCTA) has shed light on the dynamics of the choroidal vasculature in AMD patients in vivo. Significant changes in choriocapillaris flow patterns and vessel density in both early and severe AMD have been found in recent OCTA investigations [63]. These findings may be related to underlying pericyte dysfunction. The potential of choroidal vascular alterations as biomarkers for AMD progression and treatment response is highlighted by these findings. Additionally, new data points to the possibility that pericytes regulate RPE function and preserve photoreceptor health in the context of AMD. According to recent in vitro research, co-culturing RPE cells with pericytes improves the function of the RPE barrier and encourages the production of important RPE-specific genes [64]. Furthermore, it has been demonstrated that pericyte-derived substances shield RPE cells from the damage brought on by oxidative stress, which is a crucial step in the pathophysiology of AMD [65]. These results underscore the intricate relationship that pericytes, the choroidal vasculature, and the RPE have in preserving retinal homeostasis, and they imply that pericyte failure could play a role in several facets of AMD pathophysiology. Interest in creating pericyte-targeted treatments has increased, since pericytes are now understood to be significant actors in AMD. Several strategies have been investigated in recent preclinical research, such as the development of cell-based therapeutics targeted at reviving or replenishing the pericyte population in the aging choroid and the use of small-molecule inhibitors to modify pericyte–endothelial cell interactions [66]. A small-molecule inhibitor of vascular endothelial protein tyrosine phosphatase (VE-PTP), which negatively regulates Tie2 receptor activation in endothelial cells, has demonstrated promise in lowering aberrant angiogenesis in a mouse model of CNV. While the drug does not act directly on pericytes, improved Tie2 signaling stabilizes endothelial function and indirectly supports pericyte–endothelial interactions [67]. Furthermore, early research has shown that stem cell-derived pericytes can integrate into the host vasculature in animal models, suggesting that these cells may have therapeutic potential for AMD [68]. Additionally, as a potential treatment for AMD, new studies have investigated pericyte-specific signaling networks. A promising target has emerged: the Notch signaling system, which is essential for pericyte–endothelial interactions. Recent findings indicate that this pathway also contributes to the developmental generation of pericytes from neural crest-derived progenitors. Together, these findings suggest that Notch signaling plays a dual role, guiding pericyte differentiation during development and supporting pericyte maintenance and function in adult vascular homeostasis [69]. In a mouse model of AMD, a recent study showed that pharmacologically activating Notch signaling in pericytes could improve their lifespan and stabilize choroidal arteries [70]. Similarly, it has been demonstrated that altering the transforming growth factor-β (TGF-β) pathway, another important regulator of pericyte activity, may help to lessen the vascular alterations associated with AMD [71].

A recent study has directly implicated choroidal pericytes as key effectors in smoking-induced vascular pathology in neovascular AMD. The authors showed that pericytes respond to cigarette smoke exposure by upregulating PlexinB1 and undergoing cytoskeletal remodeling and transdifferentiation into a contractile, myofibroblast-like phenotype. This pericyte activation disrupted endothelial interactions and promoted choroidal neovascularization. Targeting the Sema4D–PlexinB1 axis mitigated pericyte dysfunction and restored vascular stability, highlighting pericytes as active drivers of neovascular changes in environmentally aggravated AMD [72].

The growing body of knowledge regarding pericyte biology in AMD makes it more evident that these cells are both an important part of the disease’s pathophysiology and a potentially effective target for therapy. The development of more complex in vitro and in vivo models that better capture the intricate pericyte–endothelial–RPE interactions, the clarification of the molecular mechanisms underlying pericyte dysfunction in AMD, and the transition of pericyte-targeted therapies from preclinical studies to clinical trials are some of the future research directions. By expanding our understanding of pericyte biology in relation to AMD, we might find novel approaches to the debilitating eye disease’s early detection, avoidance, and treatment.

## 5. Glaucoma and Pericyte Involvement

Globally, glaucoma is the primary cause of permanent blindness. Glaucoma is a collection of visual neuropathies characterized by progressive retinal ganglion cell (RGC) death and optic nerve degeneration. Although increased intraocular pressure (IOP) is a major risk factor, there is still much to learn about the precise mechanisms driving glaucomatous damage. The possible involvement of pericytes in the pathophysiology of glaucoma has been clarified by recent studies, especially in relation to neurovascular coupling and ocular blood flow regulation [73]. A sophisticated network of capillaries that are closely linked to pericytes extensively vascularizes the optic nerve head (ONH), a crucial site of damage in glaucoma. It has been demonstrated that these ONH pericytes are essential for preserving the integrity of the blood–brain barrier and controlling local blood flow in response to metabolic demands [74].

Recent data points to the possibility that pericyte dysfunction has a role in the vascular anomalies linked to glaucoma. Reduced capillary density and increased tortuosity are two notable changes in the retinal and ONH microvasculature of glaucoma patients found in recent investigations employing adaptive optics scanning laser ophthalmoscopy [75]. Pericyte depletion and modified pericyte–endothelial interactions have been linked to these vascular alterations. Histological studies of glaucomatous human donor eyes have shown substantial capillary loss in the optic nerve head, particularly in regions vulnerable to early damage. Although pericytes were not directly assessed, this vascular degeneration strongly suggests a disruption of the pericyte–endothelial cell interface, which is essential for capillary stability [76].

Pericyte failure in glaucoma is caused by a variety of processes, most likely involving both vascular and mechanical aspects. It has been demonstrated that ONH tissues, such as the lamina cribrosa and peripapillary sclera, are mechanically stressed by elevated IOP, a characteristic of numerous types of glaucoma. Mechanical strain can directly affect pericyte function, changing their contractile characteristics and the production of important signaling molecules, as recent in vitro investigations have shown [77]. In ONH tissues, prolonged mechanical stress has also been linked to elevated oxidative stress and inflammation, which may jeopardize pericyte survival and function even further [78].

Pericytes are essential to the pathophysiology of glaucoma, as vascular dysregulation has been linked to the disease in addition to mechanical causes. Recent studies have demonstrated the critical role neurovascular coupling—the process by which localized variations in blood flow are brought about by brain activity—plays in preserving RGC health. In a study employing two-photon microscopy in a glaucoma-stricken animal model, decreased neurovascular coupling in the retina was linked to altered pericyte-mediated capillary dilatation and constriction [79]. This blood flow dysregulation may cause oxidative stress and episodes of localized hypoxia, which may increase the sensitivity of RGCs in glaucoma.

Researchers noted increased vascular leakage and the buildup of serum proteins in the retina in a rat model of experimental glaucoma along with a marked decrease in the pericyte covering of retinal capillaries [80]. These results raise the possibility that the inflammatory and neurodegenerative processes linked to glaucoma may be facilitated by pericyte-mediated BRB degradation.

A possible function for pericytes in regulating the glial response in glaucoma is also suggested by emerging data. One typical hallmark of glaucomatous optic neuropathy is reactive gliosis, which is defined by the activation and proliferation of Müller cells and astrocytes. Complex relationships between pericytes and glial cells in the ONH and retina have been uncovered by recent investigations. It has been demonstrated that human retinal pericytes, when exposed to inflammatory stimuli, can secrete pro-inflammatory cytokines and proteases—such as IL-1β, IL-6, CCL2, VEGF, and MMP-9—that promote astrocyte activation and migration, potentially contributing to extracellular matrix remodeling in the glaucomatous ONH [81]. Conversely, activated astrocytes can influence pericyte behavior through the secretion of various factors. For example, astrocyte-derived laminin has been shown to regulate pericyte differentiation and maintain blood–brain barrier integrity. Disruption of this interaction can lead to pericyte dysfunction and vascular instability, which are critical factors in disease progression [82].

The identification of pericytes as key participants in the pathophysiology of glaucoma has sparked interest in creating novel treatment approaches that specifically target these cells. Several strategies have been investigated in recent preclinical research, such as the development of small-molecule inhibitors to modify pericyte contractility and enhance ocular blood flow and the use of antioxidants to shield ONH pericytes from damage caused by oxidative stress [83,84]. Furthermore, research is being performed on cell-based treatments that try to restore or revitalize the pericyte population in glaucomatous eyes. In a rat model of ocular hypertension, a recent study showed that when injected intravitreally, pericytes generated from mesenchymal stem cells may integrate into the retinal vasculature and enhance retinal blood flow [85].

In addition, recent studies have investigated the possibility of using pericyte-specific signaling pathways as a therapeutic approach for glaucoma. A promising target has emerged: the Notch signaling system, which is essential for pericyte–endothelial interactions. Pharmacological stimulation of Notch signaling in retinal pericytes has been shown in a study utilizing a mouse model of glaucoma to improve survival of and stabilize the retinal microvasculature, perhaps maintaining RGC function [69,86]. The involvement of Notch signaling in both AMD and glaucoma highlights its potential as a common therapeutic axis in retinal vascular diseases. Similarly, glaucoma-related vascular alterations and RGC loss may be mitigated by altering the TGF-β pathway, another important regulator of pericyte function [87].

The growing body of knowledge regarding pericyte biology in glaucoma indicates that these cells are both an important part of the disease’s pathophysiology and a potential target for treatment. Future directions for research include developing more advanced in vivo imaging methods to visualize and measure pericyte function in the human eye, deciphering the molecular mechanisms underlying pericyte dysfunction in glaucoma, and advancing pericyte-targeted therapies from preclinical studies to clinical trials. By expanding our understanding of pericyte biology in relation to glaucoma, we might find novel approaches to the complicated and debilitating eye condition’s early detection, prevention, and treatment.

## 6. Retinal Vein Occlusion and Pericyte Alterations

A common retinal vascular condition known as retinal vein occlusion (RVO) is characterized by restriction of the retinal venous circulation, which can result in retinal bleeding, edema, and even vision loss. Although retinal vein thrombosis is the main pathogenic mechanism, pericytes have been shown to have a critical role in the onset and progression of RVO [88]. The complex system of retinal capillaries, which are closely linked to pericytes, experiences significant changes in RVO, which play a role in the disruption of the BRB and consequent macular edema [89].

Significant changes in the retinal microvasculature of RVO patients have been found in recent studies using the advanced imaging technique OCTA [90,91]. These changes include reduced capillary density, increased vessel tortuosity, and the presence of microaneurysms. Pericyte dysfunction in RVO is caused by a variety of processes, most likely including both inflammatory and hemodynamic variables. RVO’s increased venous pressure and decreased blood flow might mechanically stress retinal arteries, which may have an effect on pericyte survival and function [92]. Moreover, it has been demonstrated that venous blockage produces a hypoxic environment that stimulates the release of VEGF and other angiogenic factors, thereby impairing pericyte stability and function [93].

The pathophysiology of RVO heavily relies on inflammation, and current studies have demonstrated the role pericytes play in this inflammatory response. According to studies, activated pericytes can release chemokines and pro-inflammatory cytokines that aid in the recruitment of inflammatory cells and the maintenance of vascular injury [94]. Additionally, the breakdown of the BRB in RVO, which is partially caused by pericyte dysfunction, permits the entry of inflammatory mediators from the bloodstream, intensifying the inflammatory environment locally [95].

Pericytes’ function in preserving BRB integrity has received a lot of attention. Macular edema, a serious consequence of RVO, can arise because of increased vascular permeability brought on by pericyte loss or dysfunction [96]. Researchers saw increased vascular leakage and the buildup of extravascular fluid in the retina along with a marked decrease in the pericyte covering of retinal capillaries in a rat model of experimental RVO [88]. These results imply that the onset and persistence of macular edema in RVO patients may be mostly due to pericyte-mediated BRB breakdown.

Additionally, new research suggests that pericytes may play a part in controlling the angiogenic response in RVO. Although the creation of collateral arteries can help restore blood flow to places that are ischemia-prone, angiogenesis that is excessive or abnormal can result in consequences like neovascular glaucoma. Complex relationships between pericytes and endothelial cells in the regulation of angiogenesis have been uncovered by recent investigations. Pericyte-derived substances, for example, have been demonstrated to affect vessel sprouting and endothelial cell proliferation, which may play a role in the aberrant vascular expansion seen in certain RVO cases [97]. On the other hand, existing capillaries may be more vulnerable to regression due to the decrease in pericyte covering, which could be a factor in the capillary dropout seen in chronic RVO [98].

An experimental branch RVO model demonstrated that nearly 40% of capillary pericytes were lost within a single week. This pericyte dropout was associated with persistent endothelial cell apoptosis and unphysiologically high turnover, indicating vascular instability. The study also demonstrated that in the context of pericyte loss, the retinal vasculature exhibited heightened sensitivity to hypoxia and abnormal endothelial proliferation, highlighting the critical role of pericytes in maintaining capillary integrity following BRVO [88]. It is becoming more and more obvious that pericytes in RVO are both an important part of the disease’s pathophysiology and a potential target for treatment as our knowledge of their biology advances. Future directions for research include developing more advanced in vivo imaging methods to visualize and quantify pericyte function in human retinas, deciphering the molecular mechanisms underlying pericyte dysfunction in RVO, and advancing pericyte-targeted therapies from preclinical studies to clinical trials. By expanding our understanding of pericyte biology in relation to RVO, we might find novel approaches to the complicated and visually devastating condition’s early identification, prevention, and treatment.

## 7. Future Directions

One of the main challenges in advancing our understanding of pericyte involvement in retinal pathologies is the difficulty of visualizing these cells in vivo. While histological analyses of post-mortem specimens have been the gold standard, several emerging imaging techniques now offer more direct, noninvasive insights. OCTA has garnered significant attention in clinical settings due to its ability to capture high-resolution vascular maps without dye injection. By detecting motion contrast from red blood cells, OCTA can delineate capillary networks in various retinal layers. However, standard OCTA typically lacks the axial resolution to reliably distinguish individual pericytes, making it better suited for broader vascular assessments rather than single-cell analysis. Enhanced algorithms and ultra-high-resolution OCTA systems are beginning to close this gap, though practical limitations (e.g., motion artifacts, shadowing) remain [99,100].

Adaptive optics imaging, particularly AO scanning laser ophthalmoscopy, is uniquely capable of resolving microscopic vascular structures at a near-cellular level. Correcting for optical aberrations in real time, AO systems can visualize capillary walls in fine detail, allowing the identification and tracking of pericytes in vivo. This unparalleled resolution comes at the cost of greater technical complexity, higher equipment expense, and a limited field of view. Prolonged imaging sessions and the need for stable fixation also pose challenges for routine clinical application [101,102].

From a clinical relevance standpoint, a multimodal approach may be most beneficial. OCTA is more widely available and faster to perform, supporting screening or longitudinal monitoring of global microvascular changes. AO-based imaging can complement this by providing a detailed view of pericyte morphology and behavior in selected regions of interest. Such combined strategies have the potential to enhance both diagnostic accuracy and research into pericyte-targeted interventions. In addition to conventional imaging, emerging technologies such as artificial intelligence (AI) are very promising for the future of pericyte research and therapy. A recent study in diabetic retinopathy developed a machine learning model capable of distinguishing pericytes from endothelial cells in retinal vascular samples [103,104]. Automated machine learning algorithms could expedite the detection of pericyte alterations such as thinning or loss of processes and track disease progression more objectively. Integrating large image datasets with AI-driven analytics may ultimately enable earlier diagnosis, personalized treatment protocols, and real-time assessment of therapeutic response.

At the same time, the development of new biomaterials and nanocarriers with controlled release and bioengineered scaffolds allows a new possibility of targeted administration of therapeutic agents. These materials would improve cell survival, integration, and modulation of the local microenvironment. In this way, it is possible to improve the effectiveness of regenerative approaches [104].

Pericyte-targeted therapies currently fall into several categories. Gene therapy relies on the use of viral vectors—commonly, adeno-associated viruses (AAVs)—to deliver genes that enhance pericyte survival or function. A key example involves introducing angiopoietin-1 (Ang1), which activates Tie2 signaling and helps stabilize blood vessels. Preclinical studies in DR models have shown that a single intravitreal injection (for instance, AAV-Ang1) can maintain vascular integrity and visual function for several months.

This strategy offers several advantages. First, it has the potential to be a single-administration therapy, providing prolonged, cell-specific effects that could reduce the frequency of repeat injections. Second, by improving pericyte survival and stabilizing blood vessels, gene therapy holds promise for the long-term protection of vision.

However, there are also limitations to consider. Efficiently targeting pericytes in vivo poses a challenge due to their heterogeneous nature and widespread distribution. There is also a risk of immune reactions to the viral vectors used in gene delivery. Finally, once introduced, it can be difficult to control or halt transgene expression, which limits the ability to intervene if complications arise [105,106].

Pharmacological modulation involves using drugs or biologics to stabilize pericytes or prevent their loss. This includes therapies that block angiopoietin-2 (Ang2)—for example, faricimab, which also targets VEGF-A—or that boost pro-survival pathways, such as PDGF-BB and β-adrenergic/Akt signaling. Additional strategies employ antioxidants or anti-inflammatory agents that inhibit AGE–RAGE signaling, aiming to protect the retinal vasculature from damage.

One key advantage of this approach is that several of these treatments are already in clinical use or at advanced stages of clinical trials. They can also be combined with existing therapies such as anti-VEGF treatments, offering a multifaceted way to address retinal pathologies. Moreover, they allow for a variety of administration methods, ranging from systemic to local options like intravitreal injections.

However, there are some drawbacks. Most pharmacological therapies require repeated dosing—for instance, regular intravitreal injections—which can be burdensome for patients. In addition, their effectiveness may decrease in more advanced stages of the disease, and not all patients respond equally to these interventions. Resistance or side effects can also limit their overall efficacy [107,108,109,110].

Pericyte transplantation involves replacing lost pericytes with pericyte-like cells or progenitors—such as mesenchymal stem cells—to help restore microvascular integrity in the retina. Experimental studies have shown that these transplanted cells can integrate into retinal capillaries, reduce vascular leakage, and enhance retinal function.

One of the main strengths of this approach is that it directly tackles the underlying cellular deficit, thereby opening the door to genuine vascular regeneration. Moreover, the transplanted cells often secrete trophic and anti-inflammatory factors, which can promote broader retinal health and potentially slow disease progression.

On the other hand, pericyte transplantation faces several challenges. Delivering the cells to the retina and ensuring their long-term survival is technically complex. Furthermore, this therapy is costly, with additional regulatory and manufacturing obstacles that can limit scalability and accessibility. There is also a risk of adverse events, including uncontrolled cell proliferation or immune reactions, which must be carefully managed [111,112,113,114].

The table below compares gene therapy, pharmacological modulation, and pericyte transplantation approaches, highlighting their mechanisms, target diseases, advantages, challenges, and development status (Table 1).

Although promising results have been obtained in different preclinical models, translational barriers must be overcome to use pericyte-targeted therapies in clinical practice. The main challenges are the biological heterogeneity of pericytes in retinal and choroidal compartments; the current lack of appropriate delivery strategies, specifically targeting pericytes without affecting other vascular cells; and the lack of well-validated biomarkers for monitoring pericyte function in vivo. Overcoming these barriers will require advances in cell-specific delivery systems, improved imaging and biomarker platforms, and rigorous clinical validation. As new therapies based on pericytes move toward clinical use, it is important not to underestimate the consequent ethical considerations. In particular, the main concerns regard the origin of the cells, the possible risks of immunogenicity or tumorigenicity, and the need for rigorous informed consent to be provided to the patient. As these techniques approach their clinical application, unambiguous ethical supervision is essential to ensure responsible innovation.

## 8. Conclusions

Pericyte research has gained increasing attention for its critical role in ocular vascular homeostasis, providing new insights into the intricate interactions between vascular dysfunction and retinal disease progression. Pericyte dysfunction has been implicated in several vision-threatening conditions, including diabetic retinopathy, age-related macular degeneration, glaucoma, and retinal vein occlusion. Pericytes are indispensable regulators of vascular stability, neurovascular coupling, BRB integrity, and angiogenesis. Pericyte loss or dysfunction is a key early event in diabetic retinopathy, driving microvascular instability and BRB breakdown. In AMD, pericytes regulate choroidal vasculature integrity, and their dysfunction contributes to choriocapillaris atrophy and pathological angiogenesis. In glaucoma, pericytes play a crucial role in optic nerve head microvascular homeostasis, and their depletion has been linked to retinal ganglion cell degeneration. Similarly, in RVO, pericyte dysfunction exacerbates vascular remodeling, BRB breakdown, and macular edema. Given their pivotal role in ocular disease progression, pericyte-targeted therapies have emerged as a promising area of research. As our understanding of pericyte biology continues to evolve, it is becoming increasingly evident that these cells are far more than passive structural components of the microvasculature. Their roles in vascular stabilization, neurovascular regulation, and immunomodulation place them at the forefront of retinal disease mechanisms and therapeutic development. Future directions for research include developing more advanced in vivo imaging methods to visualize and quantify pericyte function in the human eye, deciphering the molecular mechanisms behind pericyte dysfunction in retinal conditions, and moving pericyte-targeted therapies from preclinical studies to clinical trials.

## Figures and Tables

**Table 1:** Overview of pericyte-targeted therapeutic strategies for retinal diseases.

Approach	Mechanism of Action	Targeted Retinal Diseases	Advantages	Limitations	Development Status
Gene therapy	Uses viral vectors (e.g., AAV) to deliver pro-survival genes (e.g., Ang1) and stabilize vascular pathways (Tie2) [67,105,106]	DR, neovascular, AMD	Potential single administration with long-lasting effects	Challenging to achieve efficient pericyte targeting; immune response risk; difficult to modulate transgene once delivered	Preclinical (animal models)
Pharmacological modulation	Drugs or antibodies to protect and reinforce pericytes (e.g., anti-VEGF/Ang2, Tie2 activators, PDGF-BB) [34,50,67]	DR, AMD, and other ischemic retinopathies	Some agents already in clinical use; can be combined with current therapies; relatively flexible dosing	Often requires repeated dosing; variable efficacy in advanced stages; possible off-target effects	In clinical use (e.g., faricimab) and in trials
Pericyte transplantation	Transplantation of pericyte-like cells or stem cell-derived progenitors to rebuild microvascular integrity [110,111,112,113,114]	DR (potentially other retinal vascular diseases)	Direct replacement of lost cells; regenerative potential	Technically complex; ensuring cell survival and function is difficult; high cost and regulatory hurdles; risk of adverse events	Preclinical to early clinical studies

DR: diabetic retinopathy; AMD: age-related macular degeneration; AAV: adeno-associated virus; Ang1: angiopoietin-1; Tie2: TEK: tyrosine kinase; PDGF-BB: platelet-derived growth factor-BB.

## Data Availability

All data are availables previous request to the authors.

## Abbreviations

The following abbreviations are used in this manuscript:

AGEs	Advanced glycation end products
AMD	Age-related macular degeneration
BRB	Blood–retinal barrier
CNV	Choroidal neovascularization
DR	Diabetic retinopathy
IOP	Intraocular pressure
MHC	Major histocompatibility complex
NF-κB	Nuclear factor-κB
OCTA	Optical coherence tomography angiography
ONH	Optic nerve head
PDGF	Platelet-derived growth factor
RGC	Retinal ganglion cell
RPE	Retinal pigment epithelium
RVO	Retinal vein occlusion
SIRT1	Sirtuin 1
TGF-β	Transforming growth factor-β
VEGF	Vascular endothelial growth factor
